# Chronic Obstructive Pulmonary Disease With Severe Airflow Limitation and Chronic Antitussive Use: Challenges in Diagnosis, Treatment Adherence, and Inhaler Technique: A Case Report

**DOI:** 10.7759/cureus.115060

**Published:** 2026-08-24

**Authors:** Pedro M Junqueira, Nuno Pina, Diogo Lobo, Renato Velhuco

**Affiliations:** 1 Family Medicine, USF Alves Martins, Unidade Local de Saúde de Viseu Dão-Lafões, Viseu, PRT

**Keywords:** antitussives, case report, copd, technique of inhaler use, treatment adherence

## Abstract

Chronic obstructive pulmonary disease (COPD) is a chronic respiratory disorder that imposes a substantial burden on patients’ quality of life, and whose effective management relies heavily on adherence to treatment and correct inhaler technique. We report the case of an 88-year-old woman with multiple comorbidities who had previously been diagnosed with asthma despite having neither undergone spirometry nor received inhaled therapy. Although she denied significant respiratory symptoms, her granddaughter reported chronic cough, wheezing, and dyspnea. Further evaluation revealed daily use of a codeine-phenyltoloxamine combination for approximately 10 years. Pulmonary function testing confirmed the diagnosis of COPD. The patient's clinical course was marked by difficulty discontinuing the antitussive medication and, following initiation of inhaled therapy, by poor adherence and incorrect inhaler use. Multiple therapeutic adjustments were required, including the introduction of a valved holding chamber, adjunctive mucolytic therapy, and escalation to triple inhaled therapy after a moderate exacerbation. Sustained symptomatic improvement and successful discontinuation of the antitussive were achieved only after repeated patient education, correction of inhaler technique, and dose adjustment. This case highlights the importance of a multidimensional approach to COPD management, emphasizing the role of patient education and empowerment, regular assessment of inhaler technique, and longitudinal follow-up, particularly in older adults and in patients dependent on non-recommended medications.

## Introduction

Chronic obstructive pulmonary disease (COPD) is a chronic respiratory condition associated with high morbidity and mortality, with a substantial impact on patients' quality of life and considerable healthcare resource utilization. Worldwide, COPD remains among the leading causes of death, and its burden is expected to increase over the coming decades, representing a major public health challenge [1]. In Portugal, data from the Burden of Obstructive Lung Disease (BOLD) study conducted in the Lisbon region estimated a COPD prevalence of 14.2% among individuals aged over 40 years [2].

Chronic obstructive pulmonary disease should be suspected in patients presenting with dyspnea, chronic cough, or excessive sputum production. Diagnosis is established by demonstrating persistent airflow obstruction on post-bronchodilator spirometry [1]. Despite the increasing number of COPD diagnoses in Portuguese Primary Health Care in recent years, the disease remains substantially underdiagnosed and consequently undertreated [1,3,4]. This is reflected in the increasing rate of COPD-related hospitalizations, according to data from the Portuguese National Observatory for Respiratory Diseases over the past five years [4].

Delayed disease recognition represents a major healthcare challenge with direct implications for disease prognosis, contributing to disease progression, exacerbations, and associated mortality [1,5]. Its causes are multifactorial, involving both patient-related factors, such as underrecognition of respiratory symptoms or attribution of these symptoms to other causes, and healthcare-related factors, particularly the underuse of spirometry in clinical practice and symptom overlap with other respiratory conditions [6,7].

Following diagnosis, COPD management may be complex, requiring symptom control, prevention and treatment of exacerbations, improvement of quality of life, and reduction of the disease’s functional impact. Current pharmacological recommendations advocate an individualized approach tailored to symptom burden, exacerbation risk, and patient comorbidities. Among nonpharmacologic interventions, optimizing inhaler technique and adherence to inhaled therapy are particularly important [1,3,5].

The aim of this case report is to describe the challenges associated with the diagnosis and management of COPD in an older patient with high clinical complexity receiving care in Primary Health Care. It highlights difficulties related to delayed diagnosis, treatment adherence, inappropriate antitussive use, and incorrect inhaler technique. Furthermore, it emphasizes the central role of the family physician in longitudinal follow-up, patient education, and optimization of disease control.

## Case presentation

An 88-year-old Caucasian woman was seen during a routine hypertension follow-up visit. She was in Stage VIII of Duvall's family life cycle [8]. She lived alone in a rural setting, maintained independence in activities of daily living, and continued to engage in agricultural work. She had informal support from her two grandchildren, who accompanied her to medical appointments. Her relevant medical history included hypertension, dyslipidemia, transient ischemic attack, and peripheral neuropathy. Her regular medications were losartan/hydrochlorothiazide 100/12.5 mg, atorvastatin 20 mg, acetylsalicylic acid 100 mg, and duloxetine 60 mg daily. She had no known drug allergies and no history of current or previous smoking. Her National Vaccination Plan was up to date for age, although there was no record of vaccination against seasonal influenza, COVID-19, or pneumococcal disease.

Review of her medical records revealed a previous diagnosis of asthma, with no evidence of prescribed inhaled therapy or documented spirometry. When questioned directly, the patient did not report any significant respiratory symptoms. However, her granddaughter described a history of chronic cough, which prompted further investigation. During this assessment, the patient reported recurrent productive cough, dyspnea on moderate exertion, and occasional episodes of wheezing, with a persistent and progressive course according to the clinical history. She denied recent hospitalizations or emergency department visits due to respiratory deterioration. Daily self-administration of a codeine-phenyltoloxamine combination for approximately 10 years without a formal medical indication was also identified. On physical examination, the patient was in good general condition, with apparently normal nutritional status and adequate hydration. Her body mass index was 20.2 kg/m², consistent with normal weight. Pulmonary auscultation revealed expiratory wheezing and bilateral basal crackles. There was no evidence of peripheral edema. Oxygen saturation on room air was 94%. Dyspnea was assessed using the modified Medical Research Council (mMRC) scale [9] and classified as grade 2. The COPD Assessment Test (CAT) [10] yielded a score of 13, suggesting a moderate symptom burden.

Given these findings, further investigation was performed to exclude the main differential diagnoses of chronic cough and exertional dyspnea in an older patient. Cardiac causes were excluded through electrocardiography, which revealed no significant abnormalities, and transthoracic echocardiography, which showed normal chamber dimensions and preserved biventricular systolic function (ejection fraction 63%), with no valvular abnormalities or pericardial effusion, making heart failure an unlikely explanation for her symptoms. Chest radiography showed no pulmonary masses, consolidation, fibrotic changes, bronchiectasis, or sequelae of tuberculosis, and the patient presented no constitutional symptoms, hemoptysis, or other red flags suggestive of malignancy. Therefore, chest computed tomography was not considered necessary at this stage. Drug-induced cough was considered unlikely, as the patient was not taking angiotensin-converting enzyme inhibitors or other medications commonly associated with cough. A diagnosis of asthma - previously attributed to the patient without spirometric confirmation - was excluded based on the late symptom onset, the absence of a documented history of atopy or symptom variability, and, decisively, the result of the pulmonary function test. Spirometry, including bronchodilator reversibility testing, demonstrated persistent airflow obstruction without significant reversibility after bronchodilation, with a post-bronchodilator forced expiratory volume in one second (FEV₁) of 45% of predicted value and a post-bronchodilator FEV₁/FVC ratio of 0.55 (Table 1). These findings, together with the chronic respiratory symptoms, confirmed the diagnosis of COPD. According to GOLD recommendations, the disease was classified as GOLD 3, Group B. Chronic exposure to household biomass combustion smoke was considered the most likely contributing factor.

**Table 1:** Pre- and post-bronchodilator spirometry results Spirometry demonstrating a post-bronchodilator FEV1/FVC ratio below the lower limit of normal, confirming persistent airflow obstruction, with an FEV1 of 45% of predicted. The bronchodilator response did not meet criteria for significant reversibility. The reference values were calculated using the Global Lung Function Initiative (GLI) equations [11]. BD: bronchodilator (salbutamol 400 µg, reassessed at 15 minutes); FVC: forced vital capacity; FEV1: forced expiratory volume in one second; PEF: peak expiratory flow; FEF 25-75: forced expiratory flow between 25% and 75% of FVC; LLN: lower limit of normal.

Parameter	Predicted	LLN	Pre-BD	% predicted	Post-BD	% predicted
FVC (L)	2.13	1.44	1.11	52	1.18	55
FEV1 (L)	1.44	0.97	0.6	42	0.65	45
FEV1/FVC	0.77	0.61	0.54	-	0.55	-
PEF (L/s)	4.06	2.02	1.95	48	2.19	54
FEF 25-75 (L/s)	0.44	-	0.13	30	0.15	34

Dual bronchodilator therapy with aclidinium bromide plus formoterol was initiated using a dry powder inhaler, and the need for discontinuation of the antitussive medication was reinforced. At reassessment two months later, the patient reported no symptomatic improvement and continued using the antitussive medication. Review of inhaler technique revealed significant difficulty using the prescribed device correctly, demonstrating that the patient was unable to use it adequately. Therefore, treatment was switched to glycopyrronium bromide plus formoterol delivered via a pressurized metered-dose inhaler with a valved holding chamber in order to reduce the technical demands of administration and better accommodate the patient's functional abilities. Despite this change, at a two-month follow-up, the patient reported little symptomatic benefit and remained reluctant to discontinue the antitussive medication. In this context, adjunctive mucolytic therapy was introduced as a strategy to facilitate gradual discontinuation of the opioid, and the risks associated with continued use were reinforced.

Subsequently, three months later, the patient attended a consultation because of worsening fatigue and productive cough. She reported mild dyspnea. At evaluation, respiratory rate was 26 breaths/min, heart rate was 90 bpm, oxygen saturation on room air was 94%, and she was afebrile (36.2°C). No significant abnormalities were identified on pulmonary auscultation or the remainder of the physical examination. Treatment adherence, assessed at that consultation through a structured clinical interview with the patient and her granddaughter regarding the daily pattern of inhaler use, was reported as adequate, and direct observation of her inhalation technique showed no critical errors. A moderate COPD exacerbation was diagnosed. A short course of oral corticosteroid therapy with prednisolone 40 mg/day for five days was initiated. Given the moderate exacerbation and the patient's blood eosinophil count of 0.45 x10^3^ cells/µL (Table 2), treatment was escalated, in accordance with GOLD 2025 recommendations, to triple inhaled therapy with budesonide plus formoterol plus glycopyrronium bromide, with salbutamol available as rescue therapy when required. Treatment was maintained using a pressurized metered-dose inhaler with valved holding chamber support. At reassessment after the exacerbation, four weeks later, sustained clinical improvement was observed, and triple therapy was continued.

**Table 2:** Laboratory findings on initial evaluation Patient's most recent complete blood count, obtained at COPD diagnosis, seven months prior to the exacerbation. The blood eosinophil count of 0.45 ×10³/µL subsequently supported the escalation to triple inhaled therapy after the moderate exacerbation, in accordance with GOLD 2025 recommendations [1]. Laboratory testing was not repeated after treatment optimization, as the patient remained clinically stable and there was no clinical indication for further blood sampling. COPD: chronic obstructive pulmonary disease

Parameter	Result	Reference range
Hemoglobin	12.7 g/dL	12.0–15.5 g/dL
White blood cells	6.6 ×10³/µL	4.0–11.0 ×10³/µL
Neutrophils	3.94 ×10³/µL	2.0–7.0 ×10³/µL
Lymphocytes	1.84 ×10³/µL	1.0–4.0 ×10³/µL
Monocytes	0.32 ×10³/µL	0.2–1.0 ×10³/µL
Eosinophils	0.45 ×10³/µL	0.02–0.5 ×10³/µL
Basophils	0.05 ×10³/µL	0.02–0.1 ×10³/µL
Platelets	318 ×10³/µL	150–450 ×10³/µL

Following a three-month interval, during a subsequent follow-up consultation, treatment adherence was reassessed through a clinical interview in which the patient and her granddaughter were asked to describe, step by step, how the inhaler was being used at home. This assessment identified underuse of the prescribed inhaler, as the patient was taking only one inhalation in the morning and one inhalation in the evening, instead of the recommended two inhalations twice daily. The dosage was corrected, and the importance of correct inhaler technique was reinforced. Adherence and inhaler technique were then re-evaluated at every subsequent consultation using the same interview-based approach, complemented by direct observation of technique. At these assessments, the patient reported progressive and significant improvement in cough, ultimately leading to complete discontinuation of the antitussive medication over the following six months.

## Discussion

Initially, this case illustrates the multiple barriers contributing to the delayed diagnosis of COPD, particularly in women and older adults. Underdiagnosis of COPD in women has been associated with the perception that it is a predominantly male disease [6-7]. Current evidence contradicts this paradigm, with recent studies conducted in several developed countries demonstrating a similar prevalence in both sexes [1]. Furthermore, advanced age and the associated clinical complexity contributed to the tendency to underestimate respiratory complaints in older adults or to attribute them to ageing or chronic comorbidities, thereby delaying the correct diagnosis [6-7]. Likewise, the absence of traditionally recognised risk factors, such as a smoking history, or the association of the disease with less conventional factors, including exposure to biomass combustion smoke (from fireplaces or wood-burning stoves), may contribute to a lower index of clinical suspicion [1,5-7]. Beyond individual challenges, these barriers also reflect shortcomings within the healthcare system, highlighting the need for continuous professional education to improve recognition of the diverse clinical presentations of COPD.

In this case, the patient had been incorrectly diagnosed with asthma for several years, without prescribed inhaled therapy or documented spirometry - a failure that reflects a well-recognised pattern [4,7]. Difficulty accessing spirometry in Primary Health Care frequently limits the objective confirmation of airflow limitation, favouring inaccurate diagnoses and undertreatment. Therefore, it is essential to promote the systematic use of spirometry whenever chronic respiratory symptoms are identified, in order to ensure an accurate diagnosis and appropriate treatment [1,3,5].

One of the most relevant aspects of this case was the prolonged use of a codeine-phenyltoloxamine combination without a formal medical indication. The use of opioids as antitussive agents in COPD is not recommended, as they have not demonstrated additional benefit and are associated with a less predictable therapeutic response and adverse effect profile [1,5,12]. Moreover, continued use contributed to suppression of the patient's respiratory symptoms, further delaying the diagnosis. The introduction of adjunctive mucolytic therapy as a transitional strategy proved to be beneficial, allowing the patient to perceive a therapeutic alternative before discontinuing the opioid. The use of mucolytics in patients with COPD is recommended and may reduce the risk of disease exacerbations [1,13].

Regarding appropriate disease management, two essential aspects should be highlighted: treatment adherence and the suitability of inhaled therapy. Treatment adherence is one of the main determinants of COPD control and, at the same time, one of the most frequent causes of treatment failure [1,3]. Consequently, poor adherence is strongly associated with an increased risk of disease exacerbations [1,14]. Family physicians should assess treatment adherence systematically and in a non-judgemental manner [3]. In this case, adherence was assessed at each consultation through structured clinical interviews involving both the patient and her caregiver, illustrating that simple, interview-based methods, particularly when complemented by collateral information from family members and direct observation of inhaler technique, can effectively uncover clinically relevant non-adherence in Primary Health Care. In this case, two distinct patterns of non-adherence were identified: continued use of the antitussive medication despite explicit medical advice to discontinue it, and underdosing of the inhaled therapy. Timely identification and correction of the latter were associated with sustained clinical improvement, demonstrating the direct impact of adherence on disease prognosis. Regarding the suitability of inhaled therapy, appropriate device selection and regular assessment of inhaler technique are essential components of effective inhaled treatment and have a well-established impact on disease control [1,5]. In this case, the initial lack of clinical improvement was partly attributable to the patient's inability to use the originally prescribed inhaler correctly. In older adults, physical and cognitive limitations, including reduced inspiratory strength, impaired coordination, and reduced comprehension, may significantly affect the ability to use certain inhaler devices [15-16]. Switching to a pressurised metered-dose inhaler with a valved holding chamber, which required less technical ability, followed by escalation to triple inhaled therapy after an exacerbation, reflected an individualized patient-centred approach consistent with national recommendations for COPD management in Primary Health Care [3,5].

Finally, the central role of the family physician in COPD management should be emphasized, not only as the prescribing clinician but also as the patient's primary physician, with a holistic understanding of the individual. The longitudinal follow-up that is unique to Family Medicine made it possible to diagnose the disease through active identification of respiratory symptoms and subsequently facilitated its management. Identification of poor treatment adherence, incorrect inhaler technique, and inappropriate medication use represents problems that would be unlikely to be detected during episodic care. Likewise, patient empowerment through repeated reinforcement of information across multiple consultations was a determining factor in achieving behavioural change. This case reinforces that effective COPD management in Primary Health Care requires time, continuity, and a patient-centred approach-core values that define Family Medicine and that are recognised by both national and international recommendations as fundamental pillars of COPD care [1,3,5].

## Conclusions

This case report illustrates the complexity inherent to the diagnosis and management of COPD in the Primary Health Care setting and highlights the importance of implementing proactive strategies for the early detection of the disease in atypical populations in future clinical practice. It also reinforces the importance of regularly reviewing the medication of older adults in order to identify potentially inappropriate medications. These measures may facilitate earlier diagnosis and timely therapeutic intervention, with a positive impact on patients' prognosis and quality of life.

The clinical improvement observed following optimization of inhaled therapy, adjustment of the inhaler device, correction of the prescribed dosage, and discontinuation of the antitussive medication highlight the impact of this approach, demonstrating that individualized management can provide meaningful benefits to patients' quality of life, even in cases of COPD with severe airflow limitation and high clinical complexity. Owing to their unique role in providing continuity and proximity of care, family physicians are uniquely positioned to identify these situations at an early stage, empower patients and their families, and coordinate truly effective longitudinal disease management.
